# Development of a Wearable Electromyographic Sensor with Aerosol Jet Printing Technology

**DOI:** 10.3390/bioengineering11121283

**Published:** 2024-12-17

**Authors:** Stefano Perilli, Massimo Di Pietro, Emanuele Mantini, Martina Regazzetti, Pawel Kiper, Francesco Galliani, Massimo Panella, Dante Mantini

**Affiliations:** 1 Department of Information Engineering, Electronics and Telecommunications, University of Rome “La Sapienza”, 00184 Rome, Italy; stefano.perilli@uniroma1.it (S.P.); massimo.panella@uniroma1.it (M.P.); 2 Comec Innovative s.r.l., Via Papa Leone XIII 34, 66100 Chieti, Italy; mantini.emanuele@comecinnovative.it (E.M.); galliani.francesco@comecinnovative.it (F.G.); 3 Healthcare Innovation Technology Lab, IRCCS San Camillo Hospital, Via Alberoni 70, 30126 Venezia, Italy; martina.regazzetti@hsancamillo.it; 4 Movement Control and Neuroplasticity Research Group, KU Leuven, Tervuursevest 101, 3001 Leuven, Belgium; dante.mantini@kuleuven.be

**Keywords:** Aerosol Jet Printing, electro-conductivity properties, impedance prediction, electromyography sensors

## Abstract

Electromyographic (EMG) sensors are essential tools for analyzing muscle activity, but traditional designs often face challenges such as motion artifacts, signal variability, and limited wearability. This study introduces a novel EMG sensor fabricated using Aerosol Jet Printing (AJP) technology that addresses these limitations with a focus on precision, flexibility, and stability. The innovative sensor design minimizes air interposition at the skin–electrode interface, thereby reducing variability and improving signal quality. AJP enables the precise deposition of conductive materials onto flexible substrates, achieving a thinner and more conformable sensor that enhances user comfort and wearability. Performance testing compared the novel sensor to commercially available alternatives, highlighting its superior impedance stability across frequencies, even under mechanical stress. Physiological validation on a human participant confirmed the sensor’s ability to accurately capture muscle activity during rest and voluntary contractions, with clear differentiation between low and high activity states. The findings highlight the sensor’s potential for diverse applications, such as clinical diagnostics, rehabilitation, and sports performance monitoring. This work establishes AJP technology as a novel approach for designing wearable EMG sensors, providing a pathway for further advancements in miniaturization, strain-insensitive designs, and real-world deployment. Future research will explore optimization for broader applications and larger populations.

## 1. Introduction

Electromyography (EMG) is a fundamental technique for analyzing muscle function by recording electrical activity generated by muscle fibers during contraction [1]. Unlike imaging methods such as MRI, CT, or ultrasound, which focus on muscle morphology, EMG provides functional insights into neuromuscular activity and motor control [1,2]. EMG sensors are widely used across various applications, including the study of upper and lower limbs and other body regions, monitoring muscle activation patterns, assessing fatigue, and diagnosing neuromuscular disorders [2].

Despite its versatility, EMG signal acquisition faces significant challenges, primarily due to air interposition at the skin–electrode interface and skin deformation during movement. These factors can cause variability in signal quality by altering local impedance and introducing noise [3,4,5]. Skin deformation, such as lateral, rotational, or vertical stretching, can further disrupt signal acquisition by changing the electron surface path and local impedance properties [3]. Gel-based electrodes reduce lateral force effects but differ from dry electrodes, which rely on skin moisture to homogenize impedance [5]. Extensive studies have been conducted on both gel-based [6,7,8,9,10,11,12,13,14] and dry electrodes [15,16,17,18,19,20,21,22,23,24,25,26,27], as well as on the influence of electrode materials, including textiles [18], polymers [28,29], and thin films [30,31,32,33]. In addition to material selection, factors like poor contact, often due to movement or hair, and the attachment method can significantly affect the skin–electrode interface [34,35]. Gels or externally applied pressure, such as tight garment bands, can mitigate these effects, but they can also distort skin properties, altering the electrode’s impedance [36,37]. These considerations highlight the importance of optimizing both electrode design and attachment methods to achieve reliable and stable EMG measurements.

In this study, we introduce a novel EMG sensor design that minimizes air interposition between the skin and the electrode, thereby reducing signal variability caused by movement and providing more accurate and stable EMG readings. We leverage Aerosol Jet Printing (AJP) technology, which offers several distinct advantages over traditional fabrication methods for the development of EMG sensors. First and foremost, AJP enables the precise deposition of conductive materials onto flexible substrates with high resolution, allowing for the creation of miniaturized, intricate circuit patterns, which are essential for wearable sensor applications [38]. The ability to print with a wide range of materials ensures compatibility with various sensor designs and functionalities. Additionally, AJP supports the fabrication of sensors with significantly reduced thickness and enhanced flexibility [39], which improves sensor integration on the skin and user comfort during movement. Our EMG sensor prototype was developed based on the CALLIBRI^®^ system [40], ensuring compatibility with existing commercial solutions. We tested the performance of the EMG sensor not only in laboratory settings but also on the skin of a participant, thereby demonstrating its practical applicability and effectiveness in real-world conditions. While our current study focuses on maintaining the scaling ratios without miniaturization, the design principles established here pave the way for future miniaturization and integration into various wearable technologies.

This article is structured as follows. In Section 2, we describe the methods used in our study, including the experimental setup, the CALLIBRI^®^ system, and the design process of our new EMG sensor utilizing AJP technology. Section 3 presents the results of our experiments, providing a detailed analysis of the frequency–impedance characteristics of both the existing commercial sensors and our newly developed sensors. Additionally, we discuss the performance comparisons and the implications of our findings. Section 4 offers a comprehensive discussion of the advantages of the AJP technique and the potential applications of our sensor design in human movement analysis. Finally, Section 5 concludes this paper with a summary of our contributions, the significance of our results, and future directions for research in this field.

## 2. Methods

### 2.1. EMG System

The experiment is based on an acquisition system on the market that is known as CALLIBRI^®^ [40]. Through the sensors with which this instrument is equipped, it is able to acquire EMG (muscle tension), which is recorded via wireless, self-adhesive sensors placed on the muscles that are to be monitored. The sensor alone does not have the capacity for wireless signal transmission; instead, an acquirer is directly connected to it, which then transfers the data to a personal computer. The EMG sensor integrated into CALLIBRI^®^ is produced with Printed Circuit Board (PCB) manufacturing techniques, which allow modest thicknesses and sufficient comfort while ensuring stretchability and flexibility. Since we designed the new sensor for compatibility with the CALLIBRI^®^ platform, we conducted a detailed analysis of the geometry of the original sensor. We started from the electrical operating conditions of the sensor on the market, which are given in Table 1. Through these characteristics, a pre-sizing was evaluated, considering that the stimulation pulse durations do not exceed 200 µs with maximum currents of 100 mA and a potential of 600 µV. The powers at play on the sensor branches are in the order of 60 µW/µs. Considering that the circuit elements have been deposited in silver and the minimum achievable section is 10 µm × 10 µm, a value equal to 100 µm is therefore obtained. The section is called upon to dissipate a power of 120 µW. The electro-conductive track has been made of Silver METALON^®^ JS-A426 (NovaCentrix, Austin, TX, USA) in the liquid aggregation state with nanoflakes finely dispersed in the solution. For the solvent, deionized water has been placed inside the atomizer. Since the resistivity of the electroconductive material depends on the curing temperature, the appropriate value for the process must be chosen. A temperature of 100 °C has been set, suitable for the application, and this corresponds to a Volume Resistivity of 7 × 10^−4^ (Ω∙cm) [41]. According to Ohm’s second law, assuming the length of the conductor is 1 mm, we have a resistance equal to 0.07 Ω/mm.

With *R* electrical resistance in Ω, *ρ* electrical resistivity Ω∙cm, l characteristic length of the circuit element mm, and *S* conductor section µm^2^. With the characteristics described, considering Ohm’s first law with a potential of 600 µV and 100 mA, there is a resistance of 0.006 Ω/mm. Therefore, the miniaturized electro-conductive trace in the developed sensor is not affected.

### 2.2. Design and Realization of EMG Sensor with AJP

**Figure 1:**
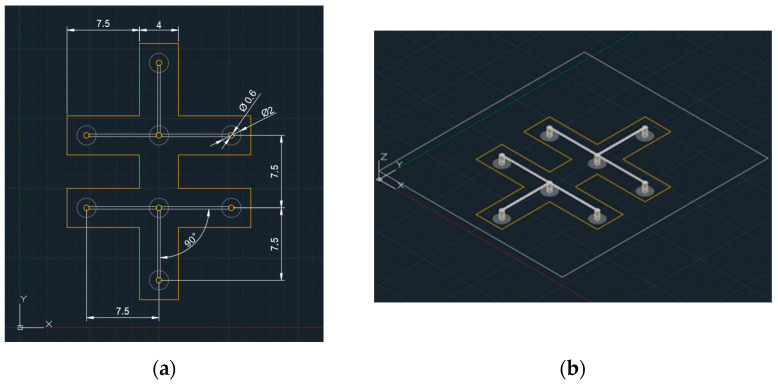
Design solutions chosen for the creation of the sensor with *AJP* technology: (**a**) eight-pole sensor in plane view; (**b**) eight-pole sensor in 3D view.

Having verified the condition of sustainability at the circuit level, we proceeded with the realization of scaled sensors on larger geometric dimensions. This was necessary to evaluate the properties of the system prior to the realization of the miniaturized traces. The CADs of the sensor design are shown in Figure 1.

Figure 1a,b shows an eight-pin sensor in the plane and 3D views, respectively. Given its geometry, the sensor is expected to have comparable structural stiffnesses along the directions of the *X* and *Y* axes but to have lower stiffness along the *Y* axis than along the *X* axis. This aspect is important because, although the sensor has a good geometric spacing of the acquisition poles, it lends itself to a preferential orientation with respect to the muscle of interest. That is, the direction of the least structural stiffness of the sensor should coincide with the preferential motion of the joint. This provides less resistance to muscle movement, making the presence of the sensor less perceptible to the participant. The possibility of realizing the sensors from AJP allows the thicknesses and, thus, the structural stiffnesses of both the base material and the electro-conductive track to be greatly reduced. It is possible to achieve overall sensor thickness values of 0.035 mm for the electro-conductive trace, i.e., the sensing component, and a thickness of 0.8 mm for the adhesive, obtaining an overall thickness of 0.835 mm. Therefore, the *AJP* technology permits us to reduce the 1.1 mm thickness of the currently marketed sensor by a value of 0.265 mm. *AJP* technology involves the deposition of multiple materials if they are in a liquid aggregate state. The process starts from an atomizer, which, in the *OPTOMEC^®^ AJ5X/FLEX* systems machine (Optomec, Albuquerque, NM, USA), can be either pneumatic or ultrasonic. The substantial difference is in the flow rate of the deposited material, with the latter providing lower flow rates but allowing fine dispersion of the particles into nanoflakes within the liquid. The case under analysis involved the use of an ultrasonic atomizer from which nanoflakes of material are carried in suspension toward the nozzle. To achieve flow focusing, a sheath gas is used, which allows the particles to converge, obtaining a trace with an accurate topology. Operationally, the machine was equipped with an ultrasonic atomizer capable of processing 2–5 µm nanoflakes. Inside the atomizer, *Silver Metalon^®^ JS-A426* electro-conductive ink and deionized water were placed, ensuring that the compound consisted of equal parts and only after thorough mechanical agitation for a time of 5 min. At the same time, a 300 µm deposition nozzle was installed, and a sand-off distance of 3 mm was set. The tests were carried out with all machine head components perfectly clean and re-installed after the cleaning cycle, as recommended by the *OPTOMEC^®^* manual. The substrate consisted of *Kapton 100HN Sheets* 304 mm × 200 mm with thicknesses 0.025 mm. From the authors’ experience in using the machine, as well as from the literature [42], some test traces used for flow stabilization were deposited in the nozzle before the deposition of the specimens for 15 min. For the setup of the printing system, the cleaning of the atomizer and the printer nozzle were verified to guarantee maximum repeatability of the process. Subsequently, pressure tightness tests were carried out, and the laboratory environmental parameters were stabilized. To allow the replicability of the electro-conductive trace, the characteristic process parameters of the machine are reported in Table 2.

## 3. Results

### 3.1. AJP Characteristics

To compare the performance of the sensor on the market with the designed one, it was necessary to proceed with the deposition of the circuit element on Kapton^®^. Since *AJP* is an additive technique, to obtain a valid deposition in terms of trace, both the material and sheath flow were optimized by calibrating the feed rates of the part/nozzle holder table and characterizing, with multiple tests, the curing curve. Table 3 shows the basic characteristics of the trace, deposition, and related curing parameters.

AJP is an additive technology in electronics that boasts the possibility of designing the electro-conductive element with sections in the order of 20 µm. Unlike conventional technologies, such as photolithography, it does not require the use of dedicated production jigs, which, although they allow significant mass production, have the limitation of being very expensive and requiring re-engineering for each layout change of the electro-conductive track. For this reason, the AJP allows full customization of the component both in geometric terms and materials, given the versatility of the printing system with respect to different types of inks. Furthermore, the deposition technology allows the manufacturing of an electrically conductive track on supports of a generic form and surface roughness, thus guaranteeing geometric precision and adhesion on human skin.

### 3.2. Lab Testing Setup

**Figure 2:**
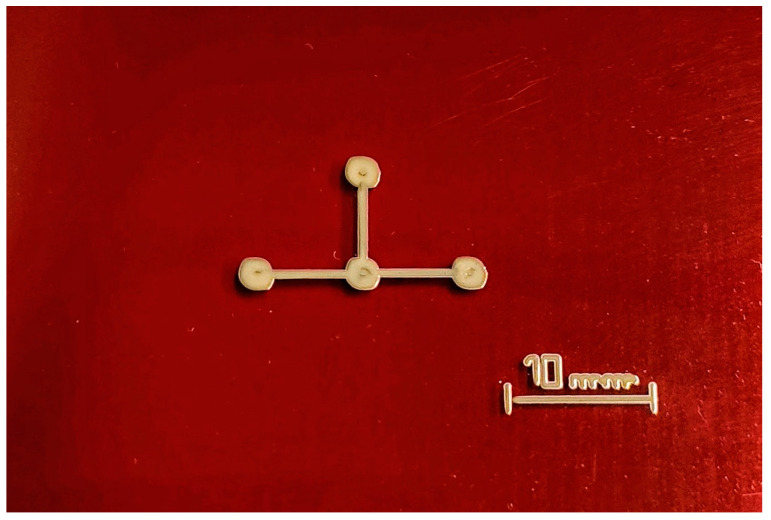
Sensor deposed with AJPs onto a Kapton^®^ sheet.

From these values, the test sensors needed for the comparison test were produced. Pictures of the sensors prepared for testing are shown in Figure 2. All connections were made with solder paste Silver Conductive Epoxy Adhesive 8331D™ from MG Chemicals Ltd. (Burlington, ON, Canada). Figure 2 shows only one semi-part of the complete sensor, as depicted in Figure 1a,b. Due to the symmetry of the sensor, a semi-part is sufficient for electrical characterization.

### 3.3. Impedance Analysis

**Figure 3:**
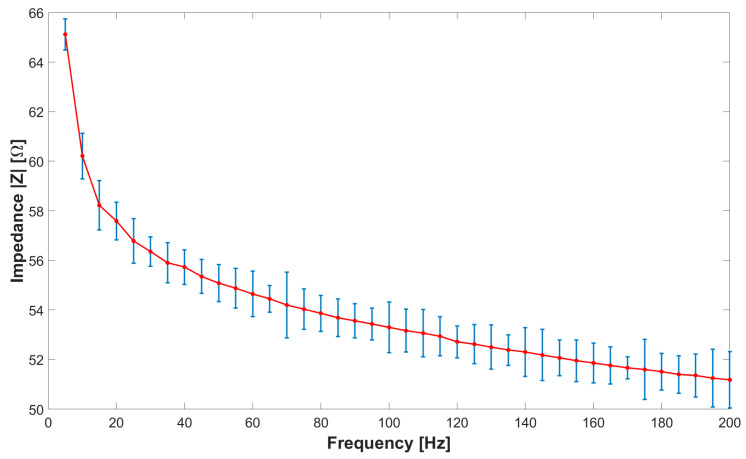
Frequency–impedance trend for the CALLIBRI^®^ sensor with conductive gel. The average and standard deviation for each set of measurements are shown.

**Figure 4:**
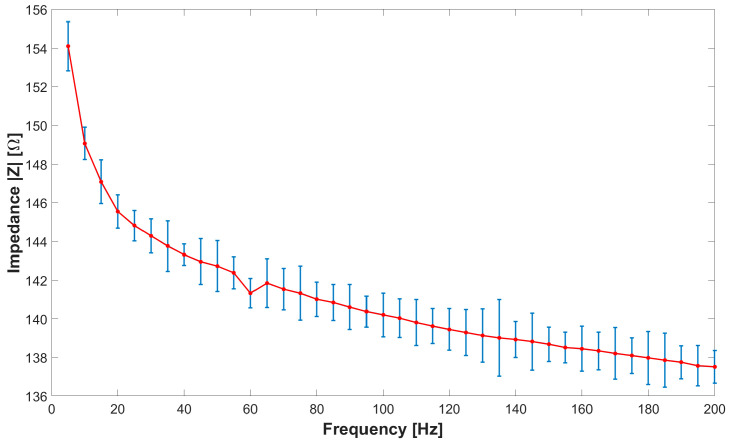
Frequency–impedance trend for the AJP sensor without conductive gel. The average and standard deviation for each set of measurements are shown.

The characterization process involves evaluating the impedance of the sensor on the market, namely, CALLIBRI^®^, as a function of frequency within a range of from 0 Hz to 200 Hz. To achieve this goal, the sensor was subjected to 40 separate tests, each at intervals of 5 Hz, to plot a characteristic curve that relates sensor impedance and operating frequency. The measurement was carried out using the volt-amperometric method, more precisely, with the downstream voltmeter, as reported in [42], the wiring diagram of which was equipped with the following instrumentation: variable frequency function generator brand RIGOL model DG2102 (Rigol Technologies Co., Ltd., Suzhou, China), voltmeter brand OWON model XDM2041 (OWON Technology Inc., Zhangzhou, China), and an ammeter brand OWON model XDM2041. The impedance *Z* of the sensor was calculated indirectly through Ohm’s law. To evaluate the new sensor against the specifications of the sensor already on the market, measurements were carried out with a similar logic as before. The tests included an analysis of the CALLIBRI^®^ sensor with contact gel when a voltage of 5 V is applied. Figure 3 and Figure 4 show the multiple frequency–impedance trends at different values of excitation potential for both the CALLIBRI^®^ and AJP sensors, respectively.

**Figure 5:**
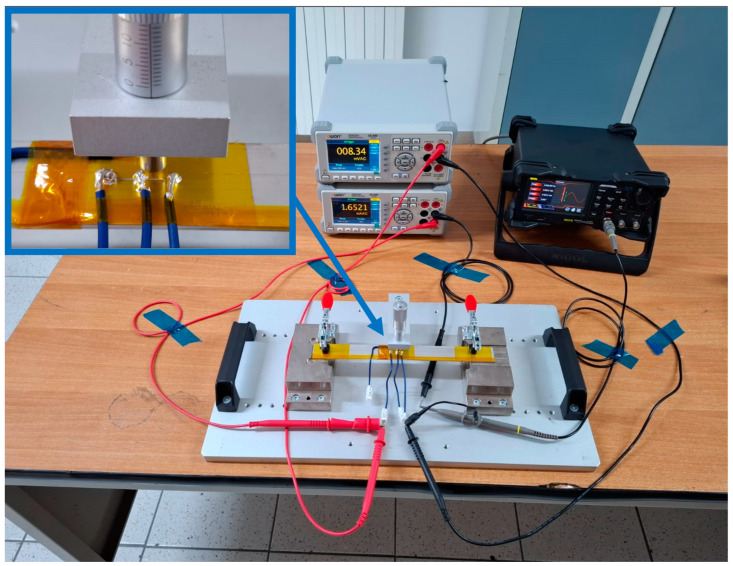
Experimental setup for analyzing the deformation/impedance properties for the sensor realized with the AJP technique.

Figure 4 shows a decreasing trend of the impedance value as the excitation frequency of the sensor increases. In this case, the AJP sensor is in a structurally undeformed condition. It is evident that the general trend is very similar to that shown in Figure 3 for the CALLIBRI^®^ sensor. Figure 5 shows an experimental set-up built specifically to test the possible interactions between the controlled structural deformation of the sensor deposed in AJP and the impedance properties. The sensor structure realized by Silver nanoparticle deposition is closely related to the Kapton substrate containing it. The electro-conductive trace, unlike an ordinary metal filament, cannot have its own volume without a support and, for this reason, cannot be tested separately from the support. In order to verify the changes of impedance under deformation, a specific support was made with the aptitude to deform a sensor placed on Kapton attached to an aluminum sheet. This sheet has dimensions of 250 × 25 × 1.5 mm and is constrained by means of two clamps (the clamps are shown in red) at the ends. In the center, the plate is stressed by a graduated micrometer screw to assess the imposed deformation. Given the natural curvature of the muscles where these sensors will be used, it was decided to impose a static deflection of 1 mm on the slab to obtain a curvature similar to real operating conditions. The setup is equipped with a voltmeter and ammeter mentioned above and the signal generator RIGOL^®^ DG2102 100 MHz 250 MSa/s. Looking at Figure 5, it can be seen that the signal generator acts on only one branch of the three constituting the sensor. In particular, the branch of interest is indicated by the red arrow on the enlargement. In order to assess the impedance with structural deformation along the X- and Y-directions (shown in Figure 1), two sensors were placed, one horizontally and one vertically.

**Figure 6:**
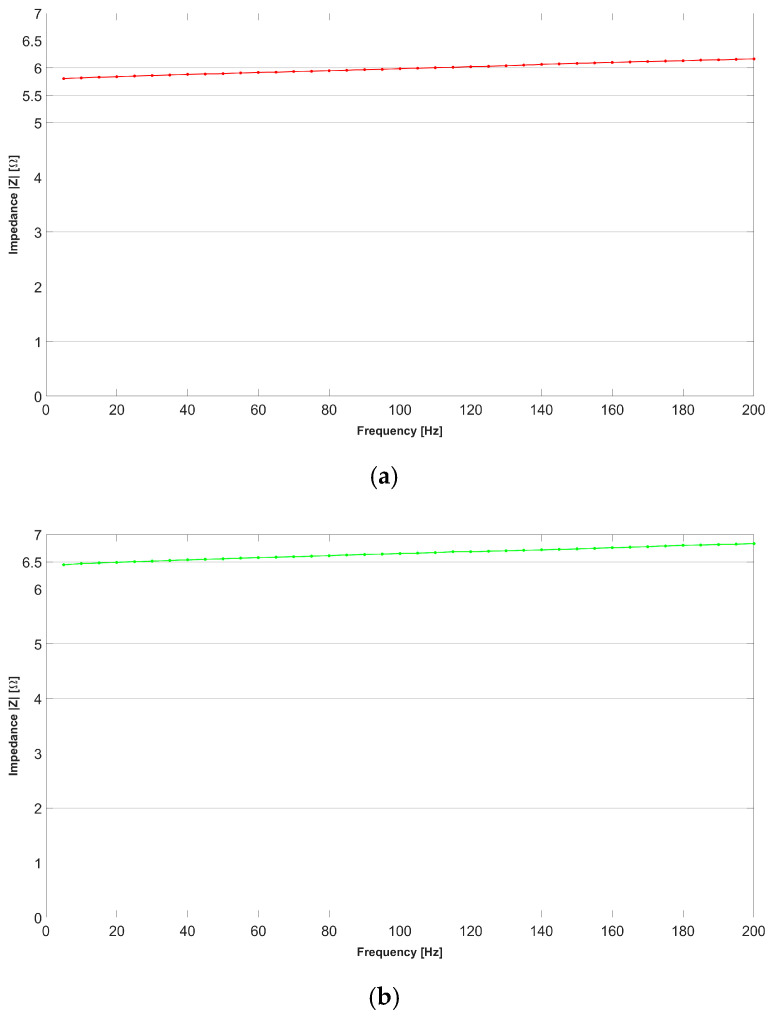
The impedance–frequency trend for the AJP sensor with the *X*-axis oriented parallel to the long side of the plate and glued to an aluminum bar. (**a**) Trend of the sensor in undeformed bar condition; (**b**) trend of the sensor in deformed bar condition.

Figure 6 shows the impedance–frequency trend measured in one branch of the sensor for the unloaded and loaded conditions, with the sensor oriented parallel to the long side of the slab and laid down according to the *X*-axis. Figure 6a shows the impedance variation in relation to the excitation frequency for the mechanically unstressed sensor. A modest increase in the impedance value can be observed as the frequency increases. This result can be found in any electrically conductive component as frequency increases. Figure 6b shows the impedance variation in relation to the excitation frequency for the mechanically stressed sensor. The trend is largely similar to the unstressed condition, but the average impedance is relatively higher. This implies that a major deformation of the sensor does not change the law of impedance variation but only generates a linear scaling effect.

**Figure 7:**
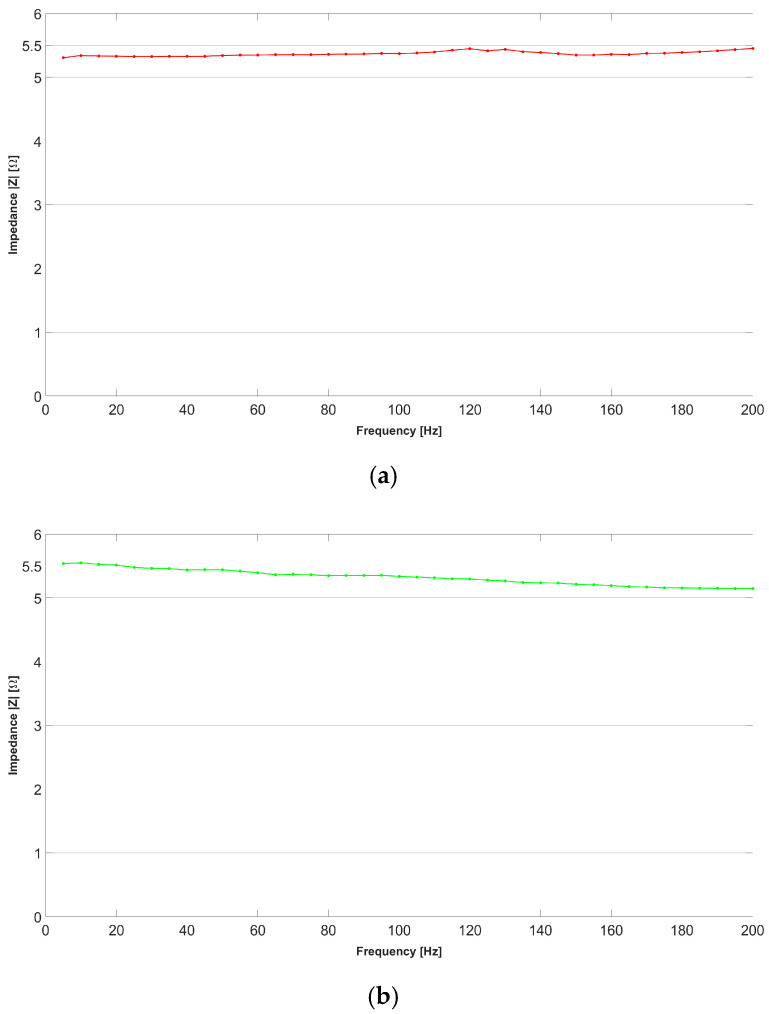
Impedance–frequency trend for the AJP sensor with the *Y*-axis oriented orthogonal to the long side of the plate and glued to an aluminum bar. (**a**) Trend of the sensor in undeformed bar condition; (**b**) trend of the sensor in deformed bar condition.

Figure 7 shows the impedance–frequency trend measured in one branch of the sensor, as described above, for a respectively unloaded or stressed slab but with the sensor oriented orthogonally to the long side of the slab and laid along the *Y*-axis. Figure 7a shows the impedance variation in relation to the excitation frequency for the mechanically unstressed sensor. A modest increase in the impedance value is observed as the frequency increases and is slightly fluctuating. Figure 7b shows the impedance variation in relation to the excitation frequency for the mechanically stressed sensor. It is possible to observe that the impedance increases as the excitation frequency increases. This is attributable to the fact that the lateral compression of the sensor modifies the impedance. However, it remains observable that, in both deformed and undeformed conditions, the sensor has an impedance response that is almost stable across frequencies.

### 3.4. Physiological Measures

**Figure 8:**
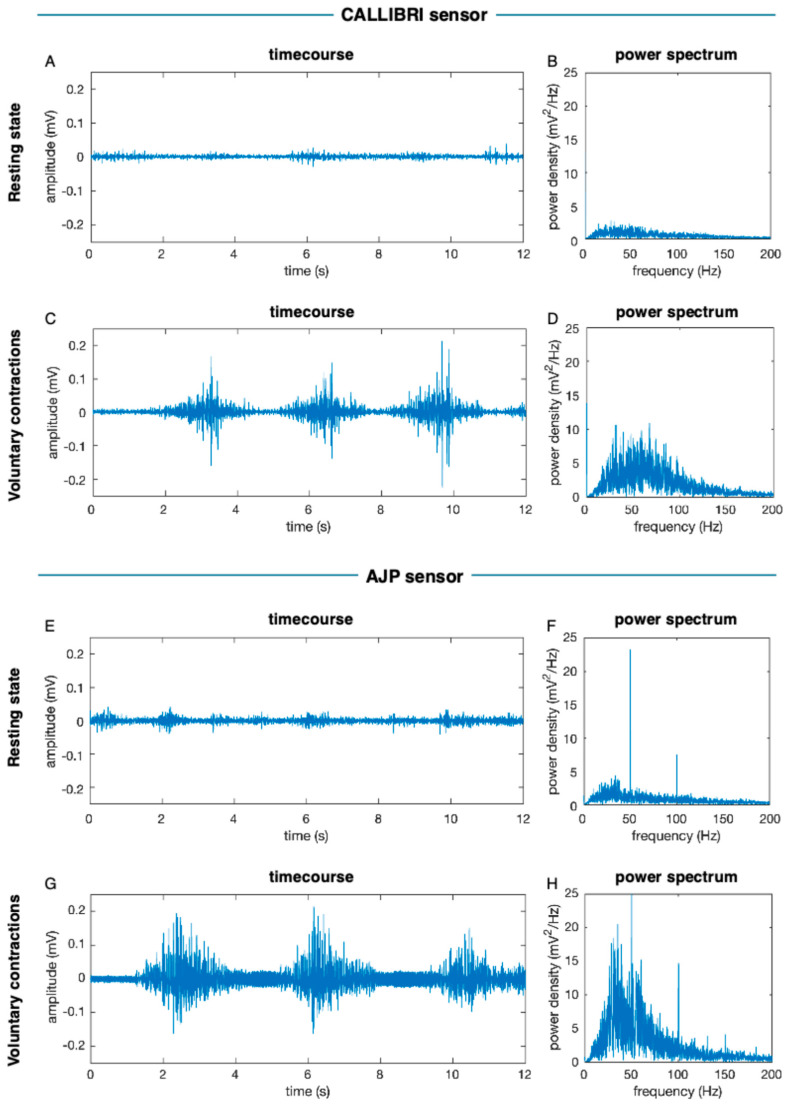
EMG signal throught the Callibri and AJP printed sensor; (**A**) Callibri resting state timecourse; (**B**) Callibri resting state power spectrum; (**C**) Callibri MVC timecourse; (**D**) Callibri MVC power spectrum; (**E**) AJP sensor resting state; (**F**) AJP sensor power spectrum; (**G**) AJP sensor MVC resting state; (**H**) AJP sensor power spectrum.

Finally, we tested the wireless EMG sensor realized with AJP technology on a healthy young participant to verify that physiological information about muscle contraction could be effectively captured. Figure 8 shows the collected EMG signal from the right biceps brachii during rest and maximum voluntary contraction, respectively, using either the CALLIBRI^®^ sensor or the AJP sensor. As expected, differences between conditions are evident in the frequency spectrum, with larger power in the maximum voluntary contraction (MVC) condition. This confirms the possibility of extracting physiologically relevant and condition-specific information from the new EMG sensor.

The testing procedure involved attaching the EMG sensor to the participant’s skin above the biceps brachii muscle. The sensor was calibrated to ensure optimal signal acquisition, minimizing noise and artifacts. During the rest condition, the participant was asked to relax their arm completely, resulting in a baseline EMG signal with minimal activity. Conversely, during the MVC condition, the participant performed a series of isometric contractions at full effort, which significantly increased the EMG signal amplitude and power.

The results provide a clear distinction between the resting state and MVC. In the resting state, the 12 s EMG trace (Figure 8A,E) displays low-amplitude signals with sparse frequency components, as illustrated in the corresponding power spectrum (Figure 8B,F). During MVC (Figure 8C,G), the EMG trace exhibits higher amplitude spikes corresponding to muscle contractions, and the power spectrum (Figure 8D,H) shows a notable increase in power across a broader range of frequencies.

This experiment demonstrates the sensor’s ability to accurately capture and differentiate between low and high muscle activity levels. The sensitivity and specificity of the AJP-printed sensor in detecting subtle changes in muscle activity make it a promising tool for various applications, including clinical diagnostics, rehabilitation monitoring, and biofeedback training. Furthermore, the wireless nature of the sensor enhances its practicality for real-world use, allowing for unrestricted movement and continuous monitoring in everyday environments.

## 4. Discussion

### 4.1. Technological Advancements

The findings from this study underscore the potential of AJP technology to transform the design and functionality of EMG sensors. By minimizing air interposition at the skin–electrode interface, the proposed sensor significantly reduces signal variability and motion artifacts, critical challenges in traditional designs. The use of AJP allowed precise control over material deposition and geometry, resulting in a thinner, more flexible, and wearable sensor that adapts seamlessly to the skin’s surface.

The comparison with commercial sensors revealed that our AJP-fabricated design maintains impedance stability across a wide frequency range, even under mechanical stress. This strain-insensitive behavior is essential for applications involving dynamic movement. Additionally, the enhanced flexibility and reduced stiffness of the sensor improve user comfort and reduce perceptibility during use.

Importantly, the physiological validation demonstrated the sensor’s ability to differentiate between resting and active muscle states with high accuracy, supporting its application in clinical diagnostics, biofeedback systems, and rehabilitation monitoring. While this study focused on a prototype design, future studies can explore miniaturization and customization for specific muscle groups. Moreover, incorporating advanced materials may enhance the sensor’s strain-insensitivity and durability, broadening its applicability.

### 4.2. Study Limitations

This study has some limitations that should be mentioned. First, environmental process variables play a fundamental role in sensor printing. These should be accurately controlled to ensure printing reproducibility. Second, additive manufacturing processes in electronics are extremely versatile but equally time-consuming and complex to optimize. The sensor produced in AJP requires hours of tests of the production process to obtain stability of the electro-conductive trace. Third, it should be considered that the sensor size and geometry may need to be optimized, depending on the target muscle. Specifically, the number of EMG sensor branches can be varied. A sensor with only two branches may not easily fit with the body geometry, whereas many sensor branches may lead to a lack of physical robustness and excessive manufacturing complexity.

## 5. Conclusions

EMG assessment in human movement analysis is rapidly evolving. Wearable sensors have the potential to capture biomechanical and physiological parameters related to training in health and performance contexts. Like all components that impact metrology, it is necessary to ensure appropriate accuracy, reliability, and repeatability. For this reason, ensuring the positional stability of the sensor over time and attempting to make it undetectable allows for optimal psychological acceptability of the sensor. In this study, we designed a new EMG sensor for fabrication with AJP. We found that the impedance–frequency trend improved for a sensor without the use of an electro-conductive gel. This allowed acquisitions at excellent sensitivity while ensuring low excitation potentials. A further reduction in footprint in terms of substrate geometry would lead to decreased stiffness, which is particularly desired for extending the range of applications.

## Figures and Tables

**Table 1:** Project parameters for a sensor on the market.

Parameter	Value
Current stimulus amplitude	1–100 mA
Stimulus frequency	1–200 Hz, step 1 Hz
Stimulating pulse duration	60, 100, 200 μs

**Table 2:** Characteristic process parameters of the AJP machine.

Parameter	Value
Sheat Gas	50 SCCM
Atomizer Gas	35 SCCM
Divert	70 SCCM
Boost	70 SCCM
UA_Heat	25 °C
BUB_Heat	25 °C
PLATEN_Heat	25 °C
Relative humidity	45%

SCCM: Standard Cubic Centimeters per Minute.

**Table 3:** Basic characteristics of the trace deposition and curing parameters.

Parameter	Value
Nozzle	300 µm
Speed of deposition	1.5 mm/s
Lenght of electroconductive trace	CAD information
Width of electroconductive trace maximize sensor	1 mm
Width of electroconductive trace miniaturize sensor	85 µm
Thickness of electroconductive trace (medium)	15 µm
Dimension of electroconductive pads	15 mm × 15 mm
Curing temperature	400.15 K
Curing time	30 min

## Data Availability

The data presented in this study are available on request from the corresponding authors.
